# Growth patterns and climate sensitivity differ for understory and canopy trees

**DOI:** 10.1093/treephys/tpag093

**Published:** 2026-07-09

**Authors:** Jakub Kašpar, Jan Krejza, Jan Holík, David Janík, Růžena Janoutová, Kamil Král

**Affiliations:** Department of Forest Ecology, Landscape Research Institute, Lidická 25/27, 602 00 Brno, Czech Republic; Department of Forest Growth and Dynamics, Global Change Research Institute of the Czech Academy of Sciences, Bělidla 986/4a, 603 00 Brno, Czech Republic; Department of Forest Ecology, Faculty of Forestry and Wood Technology, Mendel University in Brno, Zemědělská 3, 613 00 Brno, Czech Republic; Department of Forest Ecology, Landscape Research Institute, Lidická 25/27, 602 00 Brno, Czech Republic; Department of Forest Ecology, Landscape Research Institute, Lidická 25/27, 602 00 Brno, Czech Republic; Department of Forest Ecology, Landscape Research Institute, Lidická 25/27, 602 00 Brno, Czech Republic; Department of Forest Growth and Dynamics, Global Change Research Institute of the Czech Academy of Sciences, Bělidla 986/4a, 603 00 Brno, Czech Republic; Department of Forest Ecology, Landscape Research Institute, Lidická 25/27, 602 00 Brno, Czech Republic

**Keywords:** canopy position, growth rate, radial growth

## Abstract

While vertical forest structure is known to influence microclimatic conditions and tree growth, detailed knowledge of differences in seasonal growth dynamics between understory and canopy trees, especially at fine temporal scales, is still lacking. In this study, we compared growth dynamics of five temperate tree species (*Picea abies, Fagus sylvatica, Carpinus betulus, Acer campestre, Ulmus laevis*) based on sub-daily measurements from automatic band and pivotal dendrometers across four old-growth forests. Hourly growth probabilities were modelled using Generalized Linear Mixed-Effects Models (GLMMs) in relation to climatic conditions and day phases. Understory trees exhibited shorter growing seasons, accumulated fewer growing hours and displayed a less distinct diel growth rhythm than canopy trees. Nonetheless, when growth occurred, their growth rate per growing hour, normalized by stem diameter, was higher. However, overall growth remained constrained, likely because light availability limited the frequency or duration of such favorable periods, preventing consistent utilization of the buffered microclimatic conditions typical of the forest understory. The GLMMs successfully captured growth dynamics in canopy trees but performed poorly for understory trees, indicating lower dependency of their growth patterns on climatic conditions. Overall, our results show that understory tree growth is constrained primarily by limited light availability rather than by atmospheric conditions, leading to fewer growth periods that account for a larger proportion of the annual increment compared with canopy trees.

## Graphical Abstract

**Graphical Abstract:**
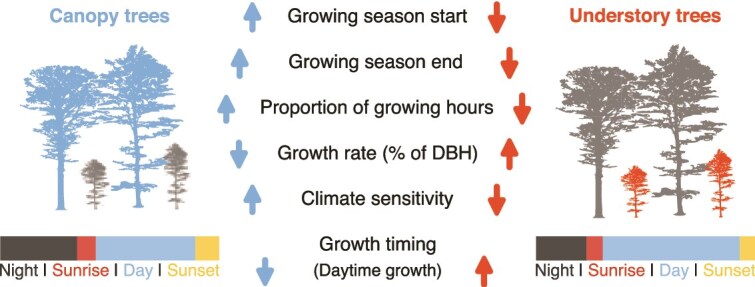

## Introduction

Forest productivity and carbon storage are closely linked to local climatic conditions and microclimatic factors within forest stands (Mo et al. 2024). Due to variations in microclimatic conditions, growth conditions vary significantly with the vertical position of a tree within the forest profile (Vinod et al. 2023). However, climate change can substantially modify these conditions by increasing temperatures, intensifying drought frequency and severity (Spinoni et al. 2018, Lewis et al. 2019) and triggering more frequent biotic and abiotic disturbances (Seidl et al. 2011, Senf and Seidl 2021). The future of forests as the largest terrestrial carbon sink (Pan et al. 2011) depends on the ability of understory (in our study, juvenile) trees to effectively cope with changing climatic and environmental conditions (McDowell et al. 2020). Therefore, understanding the growth patterns of understory trees and their link to climatic conditions is crucial for predicting the impact of the changing climate on future forest compositions.

Ontogenetically determined differences lead to variation in tree phenology among juvenile and adult trees (Vitasse 2013), as well as in the growth timing of trees at different canopy positions (Liu et al. 2018). However, in case of tree growth, the ontogeny represents only one of several interacting factors, alongside day length and climatic conditions (Huang et al. 2020, Anderson-Teixeira et al. 2022). For instance, having their apex in proximity of the ground may be both beneficial or limiting for understory trees (generally small trees) at the start of the growing season (Körner 2012, Vitasse et al. 2014). However, tree survival ultimately depends on gaining a competitive advantage by outgrowing competitors and maximizing resource acquisition (light and water) at their expense (Janík et al. 2016). Later in the growing season, the microclimate under the canopy may mitigate the negative effects of high temperatures, solar irradiance and the vapor pressure deficit (VPD) (von Arx et al. 2013, De Frenne et al. 2021) and promote cambial kinetics (the rate xylem cell differentiation) in understory trees (Cabon et al. 2020, Peters et al. 2021). In contrast, canopy trees are more directly exposed to atmospheric conditions, which may limit their growth during the same periods (Kašpar et al. 2024). On the other hand, under the canopy the limited solar radiation reduces the production of photosynthates (Messier et al. 1998, Balandier et al. 2007), constraining the carbon supply needed for tissue formation (Hartmann and Trumbore 2016, Fatichi et al. 2019) and ultimately reducing tree growth (Strieder and Vospernik 2021). Conversely, insufficient resources after foliage of canopy trees could force understory trees to reach peak growth rates before foliage development, and disrupt the growth synchronicity with upper-canopy trees. However, without direct and detailed measurements of radial growth dynamics, the differences in phenology and growth patterns cannot be fully evaluated.

Individual tree species vary in their response to climatic conditions (Rossi et al. 2006, Prislan et al. 2013, Chen et al. 2019). The number of suitable growth periods differs between species under identical climatic conditions, reflecting species-specific responses (Etzold et al. 2022, Tumajer et al. 2022, Kašpar et al. 2024). This includes the reduction of radial in favor of apical growth under limited source availability (Noyer et al. 2019), and differences in functional traits arising from variations in anatomical structure (Jandl et al. 2007, Gryc et al. 2008, 2011, Hérault et al. 2011, Mo et al. 2024), or different shade tolerance (Lichtenthaler et al. 2007, Petritan et al. 2007).

Current knowledge of differences in climate–growth sensitivity and growth patterns between canopy and understory trees is largely based on dendrochronological studies, which rely on annual growth increments (Martín-Benito et al. 2008, Mérian and Lebourgeois 2011, Carnwath et al. 2012, Martínez-Vilalta et al. 2012). Such data lack the temporal resolution required to capture intra-seasonal or diurnal growth dynamics and often fail to reflect short-term responses to environmental variability under natural conditions. The automatic dendrometers enables monitoring of stem size variation in daily (van der Maaten et al. 2018, Etzold et al. 2022, Kašpar et al. 2024) or even sub-daily scales (Peters et al. 2021, 2023, Zweifel et al. 2021, Tumajer et al. 2022, Salomón and Camarero 2025). Despite this technical advancement, these studies have focused primarily on canopy trees or on (understory) individuals with larger diameters (Strieder and Vospernik 2021). As a result, our understanding of how growth dynamics and climate–growth sensitivity differ between canopy and understory trees (in sensu our study juvenile) remains limited, particularly at fine temporal scale.

In this study, we investigate how tree growth patterns and dynamics differ between canopy and understory trees, with a particular focus on the mitigating effects of microclimatic conditions beneath the forest canopy. We hypothesize that: (i) the radial growth of understory trees peaks in the early growing season, prior to full leaf-out of canopy trees (when light availability in the understory is enhanced); and (ii) understory trees are less sensitive to atmospheric stressors, particularly elevated VPD and excessive solar radiation, than canopy trees due to the buffering effect of forest canopy.

## Materials and methods

### Study sites

This study was conducted in four old-growth forests in Czechia: Boubín, Eustaška, Ranšpurk and Žofín. In the 19th century, several noble families (at our study sites, the Schwarzenbergs, Lichtensteins and Buquoys) prohibited any management activities in order to preserve the natural character of those forests. The strict protection, furthered by the later declarations of nature reservations, allowed for spontaneous forest development for over a century and has resulted in the development of forests with diverse age and vertical structure in all sites (Král et al. 2014, 2018). Together, these sites represent characteristic natural forest types along an elevational gradient, ranging from lowland floodplain forests to the treeline in Czechia.

Eustaška (50°4′N, 17°16′E) is a Norway spruce–dominated (*Picea abies* (L.) Karst.; hereafter spruce) mountain forest in the Jeseníky Mts, located below the treeline at 1250 m a.s.l. and strictly protected since 1990. The mean annual temperature in Eustaška is 2.1 °C, and annual precipitation is 1193 mm (chmi.cz). Boubín (48°58′N, 13°48′E) is a mountain forest in the Šumava Mts, characterized by a nearly equal presence of spruce and European beech (*Fagus sylvatica* L.; hereafter beech). It is located at an elevation of 1020 m a.s.l. and has been strictly protected since 1858. The mean annual temperature in Boubín is 4.9 °C, and annual precipitation is 1067 mm (chmi.cz). Žofín (48°40′N, 14°42′E) is a beech-dominated forest with an admixture of spruce (Kašpar et al. 2021). It is located in the Novohradské Mts at 780 m a.s.l. and has been strictly protected since 1838. The mean annual temperature in Žofín is 6.2 °C, and annual precipitation is 866 mm (chmi.cz). Both spruce and beech significantly differ by their shade tolerance, when spruce is intermediately shade-tolerant conifer tree species and beech is shade-tolerant broadleaved tree species (Lichtenthaler et al. 2007, Petritan et al. 2007). Ranšpurk is a lowland forest situated in South Moravia on the Morava-Dyje floodplain (48°41′N, 16°57′E) at an elevation of 152 m a.s.l. The dominant tree species are field maple (*Acer campestre* L.; hereafter maple) and European hornbeam (*Carpinus betulus* L., hereafter hornbeam), accompanied by European white elm (*Ulmus laevis* Pall.; hereafter elm), narrow-leaved ash (*Fraxinus augustifolia* Vahl.) and small-leaved linden (*Tilia cordata* Mill.). Ranšpurk has been strictly protected for the last 90 years. The mean annual temperature in Ranšpurk is 9.9 °C, and annual precipitation is 545 mm (chmi.cz). Maple is light-demanding tree species, while elm and hornbeam are more tolerant to shade conditions (Caudullo and De Rigo 2016, Sikkema et al. 2016, Zecchin et al. 2016).

### Dendrometer data collection and preparation

**Figure 1:**
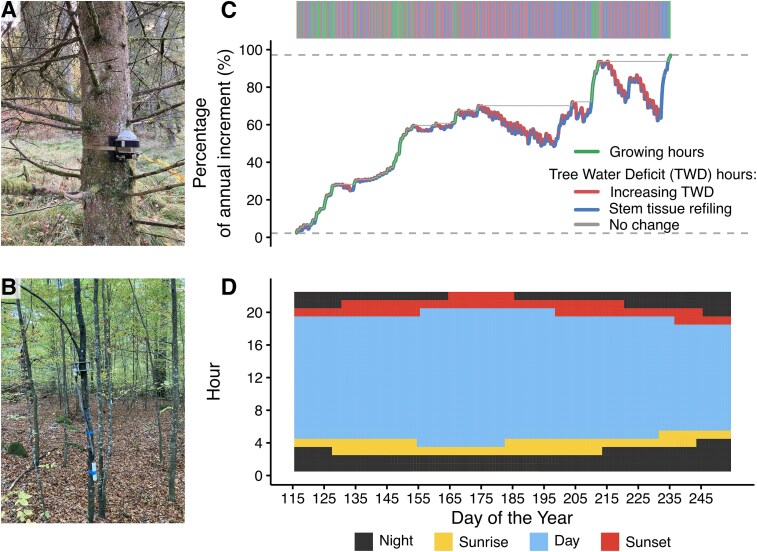
Methodological approach. (A) DRLRS26C dendrometer installed on a spruce in Žofín; (B) a PDS40P dendrometer installed on a beech in Žofín; (C) identification of growing hours (color-coded dots correspond to the tile plot), the zero-growth curve is shown as a solid gray line, and dashed gray lines indicate thresholds defining the start and end of the growing season; (D) classification of individual day phases. Photos (A and B): Jakub Kašpar.

At each site, we selected several canopy and understory trees of each dominant tree species (Table 1). We defined canopy trees as mature individuals with crowns established in the uppermost canopy layer, with a minimum diameter at breast height (DBH) >25 cm and the capability of seed production. Understory trees were defined as juvenile individuals with a height <30% of the canopy tree height and a DBH between 2 and <4 cm in the first year of measurement, showing no signs of reproduction over the entire study period. The growth of canopy trees was measured using DRL26C automatic band dendrometers (Figure 1A; emsbrno.cz), while PDS40P pivotal dendrometers were installed on understory trees (Figure 1B; emsbrno.cz). Growth data were recorded at 1-h intervals (growing hours) with a precision of 1 and 1.2 μm for DRL26C and PDS40P, respectively. Before each growing season, the health status of each tree was checked. At the end of the growing season 2024, the DBH of each tree was recorded, and a picture of the canopy cover above all understory trees was taken using a fish-eye adapter (Apexel HD 195 Fisheye), mounted on an iPhone SE 2020 and processed using the R package ‘hemispheR’ (Chianucci and Macek 2023, R Core Team 2024) to calculate canopy openness above the apex.

**Table 1:** Characteristics of measured trees. Characteristics of understory (juvenile) trees are written in brackets. Tree size characteristics correspond to the year 2024, the final year of measurement.

Site	Species	Growing seasons	Number of trees	Mean DBH (mm)	Mean annual increment (mm)
Ranšpurk	Maple	2019–24	6 (6)	516 ± 166 (53 ± 13)	2.5 ± 1.6 (2.8 ± 1.9)
Ranšpurk	Hornbeam	2019–24	6 (3)	516 ± 196 (39 ± 12)	2.6 ± 1.0 (0.3 ± 0.1)
Ranšpurk	Elm	2019–24	2 (1)	662 ± 169 (41)	5.5 ± 1.7 (1.4 ± 0.5)
Žofín	Beech	2018–24	33 (12)	545 ± 171 (28 ± 6)	3.3 ± 1.9 (0.9 ± 0.5)
Žofín	Spruce	2018–24	16 (2)	714 ± 241 (34 ± 6)	3.6 ± 1.9 (1.5 ± 0.6)
Boubín	Beech	2019–24	8 (3)	447 ± 131 (61 ± 8)	4.3 ± 2.1 (1.3 ± 0.7)
Boubín	Spruce	2019–24	9 (3)	474 ± 233 (48 ± 22)	4.6 ± 2.6 (2.4 ± 1.7)
Eustaška	Spruce	2020–24	7 (24)	498 ± 188 (55 ± 9)	2.5 ± 1.1 (3.5 ± 1.6)

Records of stem circumference variation (band dendrometers) and stem diameter variation (pivotal dendrometers) were cleaned of measurement errors (e.g., sudden jumps or tape rewinding) and were harmonized to reflect stem diameter variation (Kašpar et al. 2024). The data were then partitioned into two key processes: growth (growing hours), represented by the stem diameter increment (SDI), and water-related processes (non-growing hours), quantified as the tree water deficit (TWD). The TWD was derived using the zero-growth concept (Zweifel et al. 2016), which involves calculating a running maximum over the data and identifying subsequent maxima as increment values. Periods of TWD were further classified into three categories: (i) increasing TWD, characterized by negative differences between two consecutive hourly measurements; (ii) stem tissue refilling, where positive increments were smaller than the previous zero-growth value; and (iii) no change, referring to periods where the difference between two consecutive measurements was zero (Figure 1C).

To compare the growth data across trees of different sizes, we normalized increment data (both SDI and increasing TWD) for each tree within each growing season, calculating the cumulative SDI on a scale of 0–100%. To minimize bias from stem rehydration at the beginning or end of the growing season, analyses were limited to 95% of the annual growth (Figure 1C) by omitting the first and last 2.5% of annual increment (e.g., Salomón and Camarero 2025). Using higher thresholds (e.g., 5% and 95%) resulted in changes to estimates of the start and end of the growing season by only a few days (Figure S1 available as Supplementary Data at *Tree Physiology* Online; or Zweifel et al. 2021, Kašpar et al. 2024). Both starts of the growing season and ends of the growing seasons are in accordance with already published studies in the Central European region based on xylogenesis data (Giagli et al. 2015, Treml et al. 2015), Vaganov–Shaskin model simulations (Tumajer et al. 2017, Kašpar et al. 2021) or their combination (Tumajer et al. 2021, 2025). In addition, relative SDI (expressed as a percentage of DBH increment) was calculated to allow comparisons of growth across individuals of different sizes.

### Climate data and day phase calculations

Climatic data were available for the years 2021–24 for Boubín and Eustaška, for the years 2020–24 for Ranšpurk and for the years 2016–24 for Žofín. All stations are positioned in open gaps to capture general climatic conditions. They measured air temperature (at 2 m above the ground; in °C), relative air humidity (%; both measured using Minikin RTHi/QTHi, EMS Brno), precipitation (sum per the interval; in mm; measured using Rain gauge Pronamic Pro and datalogger Minikin ERi, EMS Brno) and global radiation (W m^−2^, using Pyranometer EMS 11S, EMS Brno). These metrics were recorded at 10-min intervals (or shorter). Meteorological data were aggregated to hourly intervals by averaging (temperature, relative humidity and global radiation) or summing (precipitation). Air temperature and relative humidity were also measured under the canopy to capture the microclimatic conditions influencing understory tree growth. These measurements were recorded at hourly intervals matching the identified growing hours.

From air temperature and relative humidity data, we calculated the VPD using the Clausius–Clapeyron formula:


\begin{align*}{e}_a=0.6108\ast{e}^{\frac{17.27\ast T}{273.3+T}}\ast \left(\frac{H}{100}\right),\end{align*}

where T is the measured air temperature (in °C) and H the relative humidity (in %) at a given time.

Since the 2021 growing season, soil water potential (SWP) has been measured using Delmhorst GB1/GB2 sensors and MicroLog SP3 data loggers (EMS Brno). Measurements were taken at 1-h intervals by a minimum of five devices per site at depths of 10 cm below ground. This depth was chosen as representative for our study because understory trees lack deep root systems, and key species such as spruce and beech have a high proportion of fine roots in this soil layer.

For each day, we determined four distinct phases: sunrise, day, sunset and night (Figure 1D). These phases were calculated using the ‘suncalc’ R package (Thieurmel and Elmarhraoui 2022) and defined as follows: day, the period when the sun is fully above the horizon; night, the period when the sun is more than 12° below the horizon; sunrise, the transition from night to day, covering the period from when the sun touches the horizon until it reaches 12° below the horizon (including nautical and civil twilight); and sunset, the transition from day to night, covering the period from when the sun touches the horizon until it reaches 12° below it (including civil and nautical twilight). If a 1-h time interval (used for growing hour calculations) spanned two phases, it was assigned to the phase where the majority of the period occurred (Figure 1D). Including nautical twilight, the durations of both transition phases (sunrise and sunset) was slightly overestimated, to get representative number of hours in these phases.

The climatic data, SWP measurements and day phases were combined for the analysis of the effect of climatic and moisture conditions on the growth of understory and canopy trees in matching hours.

### Statistical analyses

Differences in the start, end and duration of the growing season, annual increments and the number of growing hours were analyzed using linear mixed effect models (‘lme4’ R package; Bates et al. 2015). To account for interannual climatic variability and repeated measurements of individual trees in different growing season, year and tree were included as random effects. Fixed effects included tree size (when evaluated differences between understory and canopy trees of the same species or species) or tree species (when evaluated differences between the same size classes of different species). This approach allowed us to control for non-independence in the data and uneven sample sizes across canopy and understory trees, as well as variation in growing hours across day phases. To assess the robustness of our results across growing seasons, we compared results for each growing season separately (aggregation analysis) and confirmed the consistency of our results.

Differences in the ‘daily growth increments’ and ‘proportion of growing hours’ during the growing season between canopy and understory trees of a given tree species were tested using a generalized linear model (GLM) with quasi-binomial distribution. The predictors were: ‘growing season progress’ (percentage of realized annual growth or number of days from the start of the growing season) and ‘canopy position’ (understory or canopy). The difference was statistically significant when the *P*-value for the size class effect was <0.05. The full model formula for this model was:


\begin{align*} &Daily\ growth\ increment/ Proportion\ of\ growing\ hours\nonumber\\&\quad \sim Growing\ season\ progress\ast Canopy\ position \end{align*}

The effect of climate on the probability of growth was evaluated separately for canopy and understory trees of each species using GLMMs with a binomial distribution, where the response variable indicated whether a given hour was classified as a growing or non-growing phase during the growing season. Meteorological metrics relevant to tree growth were included in the model, consistent with those used in previously published studies that employed a similar modelling framework (Etzold et al. 2022, Tumajer et al. 2022, Kašpar et al. 2024):


\begin{align*} Growth& \sim Air\ temperature+ VPD+ Global\ radiation\\&\quad + Precipitation\ totals+ SWP+ Day\ length+ Day\ phase\\&\quad+ VPD: SWP+ SWP: Day\ length+ VPD: Day\ length\\&\quad+ Global\ radiation: Day\ length+\left(1| Tree\right)\end{align*}

where ‘Growth’ is binomial variable indicating whether growth occurred or not, ‘Air temperature’ represents temperature (°C), ‘VPD’ is the vapour pressure deficit (kPa), ‘Global radiation’ represents solar irradiance (W.m^−2^), ‘Precipitation totals’ refer to sum of precipitation within each hourly interval (mm), ‘SWP’ refers to soil water potential in (−bar), ‘Day length’ is the duration of daylight (decimal hours) and ‘Day phase’ is a categorical variable with four levels: sunrise, day, sunset and night. All fixed predictors were scaled, and individual trees were nested as a random effect. The analysis was conducted using the ‘lme4’ R package (Bates et al. 2015).

Residual diagnostics revealed mostly moderate positive lag-1 temporal autocorrelation within Tree and Year time series (ACF₁ ≈ −0.03 to 0.38; Table S1 available as Supplementary Data at *Tree Physiology* Online). Model overfitting was assessed using 10-fold cross-validation, wherein GLMMs were repeatedly refitted on training subsets and evaluated on held-out data using log-loss. For each model, the mean log-loss across folds was calculated as an estimate of out-of-sample prediction error. Cross-validation indicated moderate predictive ability, with mean log-loss values ranging from 0.29 to 0.48 for canopy and from 0.19 to 0.37 for understory trees. Due to the low number of trees at the Ranšpurk site (specifically of hornbeam and elm), a GLM was used instead of GLMM, using the same formula without a random effect. All models were conducted for the common period of dendrometer, climatic and SWP measurements.

The *R*^2^ of individual models was extracted using the ‘MuMIn’ R package (Bartoń 2016). Effect sizes of predictors were represented by their estimates, as all fixed predictors were scaled. All data procession and analyses were done using R statistical software 4.4.1 (R Core Team 2024).

## Results

**Figure 2:**
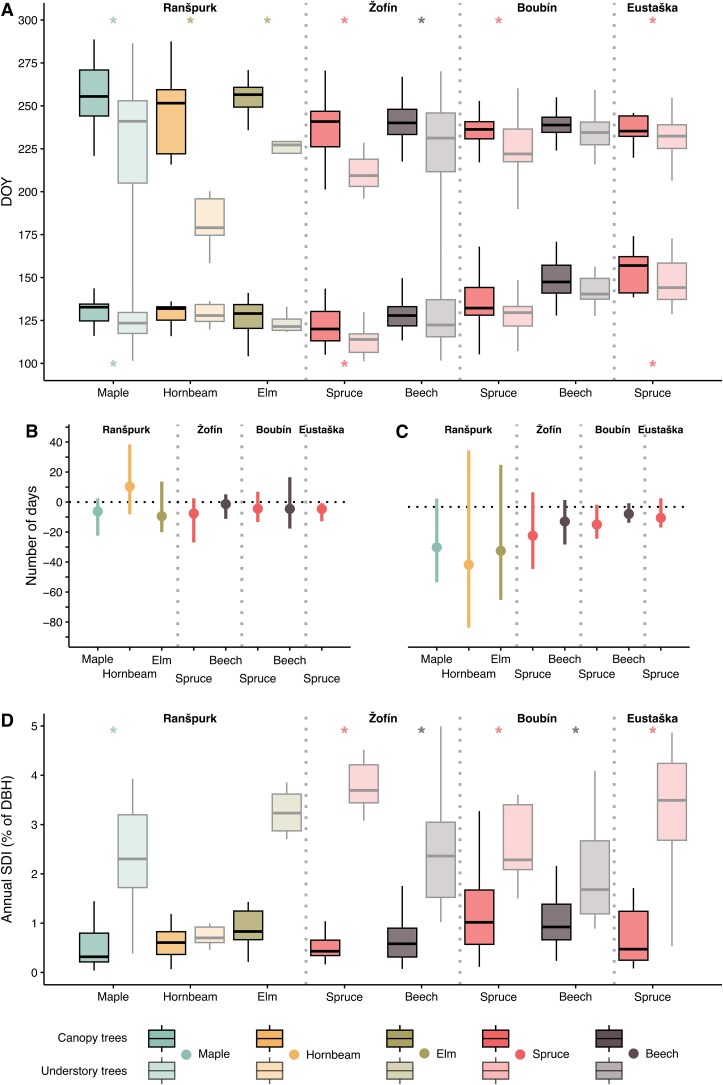
General parameters of the growing season for canopy and understory trees. (A) Starts and ends of the growing season; (B) annual stem diameter increment (SDI) in relation to the diameter at breast height (DBH) at the beginning of the growing season. The boxes represent the 25th, 50th and 75th percentiles, while the whiskers illustrate 1.5 times the interquartile range. Statistically significant differences between canopy and understory trees are marked by asterisks.

Understory trees began their growing season 5.3 ± 4.2 days earlier than canopy trees (Figure 2A and B). The significant (*P* <0.05) difference was found for spruce in Žofín and maple in Ranšpurk. Similarly, understory trees ended their growing season 19.8 ± 17.4 days earlier than canopy trees, with significant (*P* <0.05) differences observed for all species at all sites except beech in Boubín (Figure 2A and C). On average, the growing season of understory trees was 14.1 ± 18.4 days shorter, but significant differences (*P* <0.05) were found only in Ranšpurk (maple and hornbeam) and in Žofín (beech; Figure 2A). While patterns varied slightly between years (Figure S2 available as Supplementary Data at *Tree Physiology* Online), the earlier end and shorter duration of the understory growing season remained consistent.

The annual SDI of understory trees was generally significantly smaller (*P* <0.05) than that of canopy trees (1.75 ± 1.85 mm; except for maple; Table 1). Understory trees, except hornbeam and elm, showed significantly (*P* <0.05) higher relative SDI (3.1 ± 2.2%; Figure 2D). The growth dynamics affected SDI, with understory trees completing 25%, 50% and 75% of their annual SDI 5.7 ± 14.2, 9.8 ± 15.7 and 14.4 ± 15.3 days, respectively, earlier than canopy trees (Figure S3 available as Supplementary Data at *Tree Physiology* Online).

**Figure 3:**
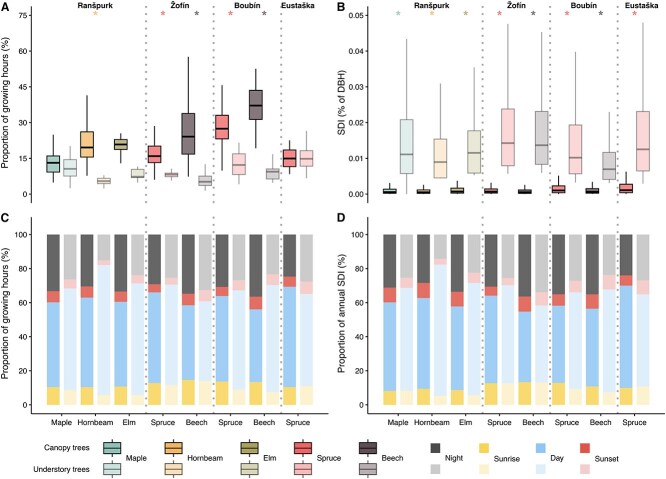
The growth patterns of understory and canopy trees. (A) Proportions of growing hours in the growing season; (B) annual increments in relation to the DBH; (C) the distribution of growing hours across different day phases; (D) the contribution of individual day phases to the annual stem diameter increment (SDI); (E) differences between the proportion of growing hours occurring in individual day phases and the proportion of day phases in the growing season; (F) differences between the proportion of cumulative SDI in individual day phases and the proportion of day phases in the growing season. The boxes represent the 25th, 50th and 75th percentiles, while the whiskers illustrate 1.5 times the interquartile range.

Understory trees completed their growth in significantly (*P* <0.05) fewer growing hours than canopy trees (5.4–15.2% vs 13.6–36.8%), except for maple and elm in Ranšpurk and spruce in Eustaška (Figure 3A). Among understory trees, spruce had more growing hours than beech at both Boubín and Žofín (ca 2.1–2.3% more). In Ranšpurk, maple had the highest proportion of growing hours (11.0%), followed by elm (8.2%) and hornbeam with the lowest (5.4%; Figure 3A). Among canopy trees, beech had the highest proportion of growing hours, with 9.3% and 9.4% more than spruce in Boubín and Žofín, respectively (Figure 3A). A similarly reversed pattern between the proportion of growing hours of understory and canopy trees was observed in Ranšpurk, where hornbeam had the most growing hours (20.7%), followed by elm 19.9% and maple 13.6%. Understory trees also had more TWD hours than canopy trees (84.8–94.6% and 63.2–86.4%, respectively; Figure S4A available as Supplementary Data at *Tree Physiology* Online). Despite relatively similar proportions of increasing TWD (32.2–36.3% and 27.0–34.7%; Figure S4B available as Supplementary Data at *Tree Physiology* Online) and stem tissue refiling hours (31.4–35.7% and 27.0–43.2%; Figure S3C available as Supplementary Data at *Tree Physiology* Online), this difference was due to significantly higher proportions of hours with no change in stem diameter in understory trees compared with canopy trees (20.3–25.0% and 5.6–10.9%, respectively; Figure S4D available as Supplementary Data at *Tree Physiology* Online). The SDI and relative SDI per growing hour was higher for understory than for canopy trees (*P* <0.05; Figure 3B and Figure S5A, B available as Supplementary Data at *Tree Physiology* Online). Similarly, increasing TWD was stronger for understory trees (Figure S5C and D available as Supplementary Data at *Tree Physiology* Online).

**Figure 4:**
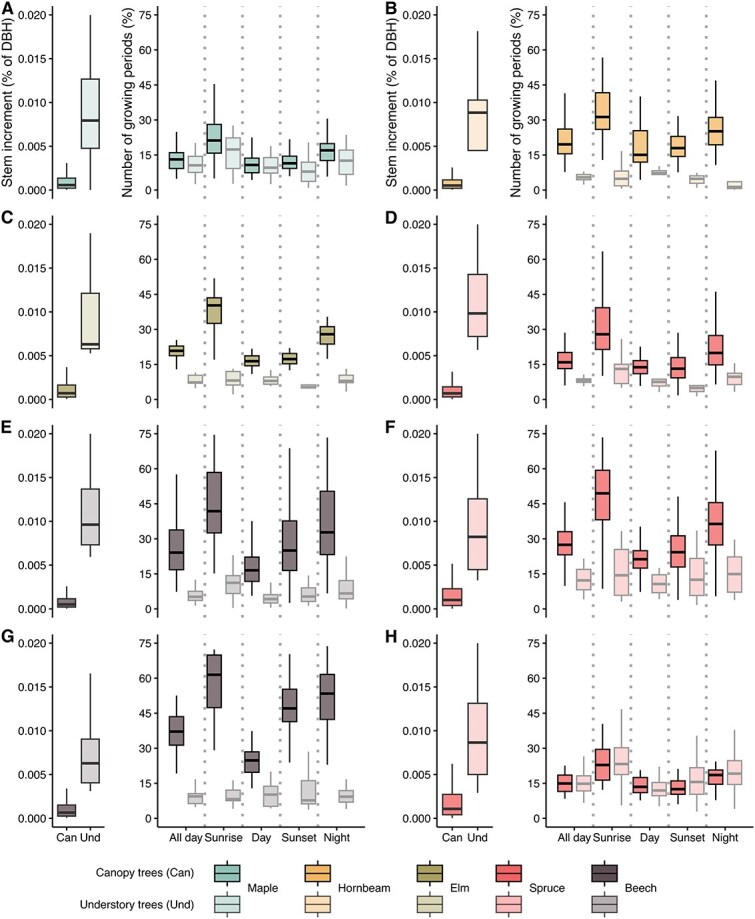
Mean growth rates and proportions of growing hours of understory and canopy trees across different day phases. (A) Maple in Rašpurk; (B) hornbeam in Ranšpurk; (C) elm in Ranšpurk; (D) spruce in Žofín; (E) beech in Žofín; (F) spruce in Boubín; (G) beech in Boubín; and (H) spruce in Eustaška. The boxes represent the 25th, 50th and 75th percentiles, while the whiskers illustrate 1.5 times the interquartile range.

Most growing hours occurred during the day (42.7–76.3%; Figure 3C), followed by night (15.1–36.5%), sunrise (5.8–14.6%) and sunset (2.8–7.4%), partially reflecting the general distribution of day phases (Figure 1D). Considering the proportions of growing hours across day phases, canopy trees had the lowest proportions of growing hours during the day (11.1–24.4%) when compared with night (17.7–58.0%), sunrise (23.7–67.0%) and sunset (12.8–49.8%; *P* <0.05; Figure 4). By contrast, understory trees showed smaller, largely non-significant (*P* > 0.05), differences across day phases (Figure 4), with the proportions of growing hours during the day 4.4–12.5%, night (2.9–20.3%), sunrise (6.4–24.3%) and sunset (4.4–16.5%). Overall growing hours were overrepresented during the night (21.8 ± 6.8% for canopy and 6.6 ± 12.4% for understory trees), sunrise (18.8 ± 5.1% and 5.0 ± 10.2%) and sunset (13.4 ± 5.1% and 1:4 ± 9.6%), and underrepresented during the day (−13.5 ± 4.2% and −2.6 ± 8.3%). This pattern was more pronounced in canopy trees compared with understory individuals (Figure S6A available as Supplementary Data at *Tree Physiology* Online).

Although, relative SDI differed significantly between understory and canopy trees, it remained relatively constant across the different day phases (Figure 4 and Figure S7 available as Supplementary Data at *Tree Physiology* Online). Consequently, relative annual SDI was generally realized during the day (Figure 3D), with a similar pattern in the over- and underrepresentation in individual day phases as observed for the growing hours. Specifically, growth was overrepresented during the night (23.1 ± 7.5% for canopy and 6.2 ± 13.3% for understory trees), sunrise (19.9 ± 5.4% and 7.7 ± 10.5%) and sunset (17.5 ± 5.0% and 5.3 ± 10.2%) and underrepresented during the day (−16.4 ± 4.5% and −5.9 ± 8.8%; Figure S6B available as Supplementary Data at *Tree Physiology* Online).

**Figure 5:**
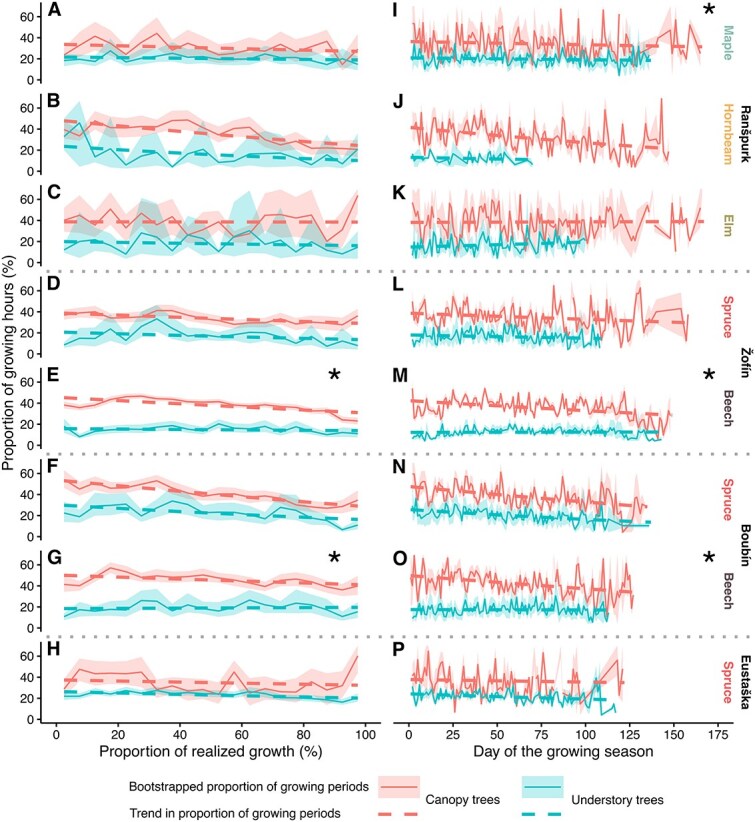
Proportions of growing hours related to realized annual stem diameter increment (SDI; left column) and to specific days of the growing season (right column). (A) Maple in Ranšpurk; (B) hornbeam in Ranšpurk; (C) elm in Ranšpurk; (D) spruce in Žofín; (E) beech in Žofín; (F) spruce in Boubín; (G) beech in Boubín and (H) spruce in Eustaška. Statistically significant differences in trends in the proportion of growing hours throughout the growing season of understory and canopy trees (*P* <0.05) are marked with an asterisk.

The proportion of growing hours generally decreased over the growing season (Figure 5). This decrease was more pronounced when related to the proportion of realized growth (Figure 5A–H) than when related to the number of days since the start of the growing season (Figure 5I–P). However, the decrease was higher for canopy than for understory trees, where it remained nearly constant. Statistically significant (*P* <0.05) differences were observed for maple (only in relation to days from the start of the growing season) and beech (at both Boubín and Žofín). Considering the growth rates over the growing season (Figure S8 available as Supplementary Data at *Tree Physiology* Online), they also remained constant or followed similar trend for both canopy and understory trees (except Hornbeam in Ranšpurk and spruce in Eustaška; *P* <0.05).

**Figure 6:**
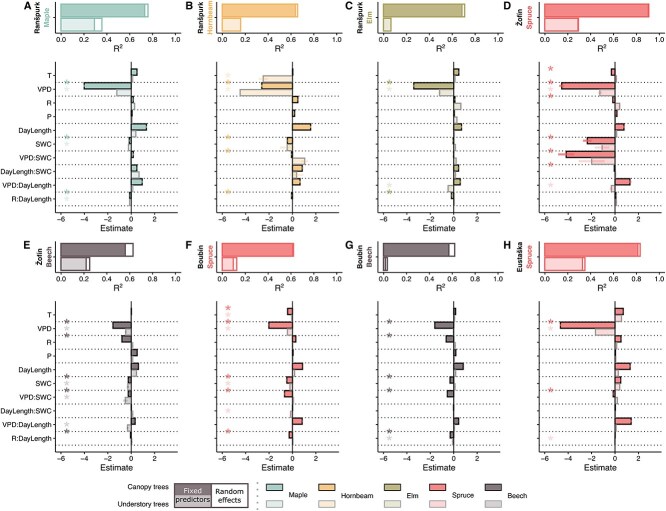
Results of generalized mixed-effects models predicting the probability of tree growth and estimates of selected meteorological predictors. (A) Maple in Ranšpurk; (B) hornbeam in Ranšpurk; (C) elm in Ranšpurk; (D) spruce in Žofín; (E) beech in Žofín; (F) spruce in Boubín; (G) beech in Boubín; (H) spruce in Eustaška. In upper barplots, the variability explained by the random effect is represented by the empty portion of the bar. Statistically significant predictors are marked with asterisks.

Models simulating the probability of a growing hour based on climatic variables and day phases explained 57.8–93.0% of the variability for canopy trees, which was more than twice the predictive power of models for understory trees (1.7–29.8%; Figure 6). Random effects explained from 0.4% to 7.0% of the variability. Among climatic metrics, VPD had the strongest effect, followed by day length and day phases (Table S1 available as Supplementary Data at *Tree Physiology* Online). The effect of global radiation was generally stronger (and positive) for understory than for canopy trees (except for hornbeam and spruce in Eustaška and Boubín; Figure 6). Apart from global radiation, the effects of climate metrics and day phases were generally stronger for canopy than understory trees (Figure 6). The effect of day (represented by the model intercept) on the probability of growth was consistently negative. In contrast, sunrise and night had mostly positive effects (except for elm and canopy maple during sunrise and for elm, canopy maple and spruce in Žofín during night). The effect of sunset differed among species and their canopy position (Table S1 available as Supplementary Data at *Tree Physiology* Online). The GLMM and GLM based on climatic metrics measured under the canopy explained less variability and produced lower effect estimates for meteorological predictors compared with models using standardized meteorological data (Table S1 and Figure S9 available as Supplementary Data at *Tree Physiology* Online).

## Discussion

This study reveals substantial differences in the magnitude of seasonal and diurnal growth dynamics and climate–growth sensitivity between understory (juvenile) and canopy (mature) trees across four old growth forests. These forests vary in climatic conditions, species composition and dominance, spanning a gradient from lowlands to the treeline ecotone. We found that the growing season of understory trees starts and ends sooner than the growing season of canopy trees, which is in accordance with phenological observations (Vitasse 2013), and highlights significant variations in growth dynamics based on tree size and canopy position (Couralet et al. 2010). More notable was the difference in growing hours, with understory trees showing fewer growing hours than canopy trees, but exhibiting higher relative SDI (Figures 2 and 4). Contrary to previous studies (Martín-Benito et al. 2008, Carnwath et al. 2012, Rollinson et al. 2021, Wang et al. 2023), which focused on climate–growth relationships using monthly or seasonal data, our approach reveals much finer-scale differences in the timing and intensity of secondary growth between canopy and understory trees. These high-resolution insights offer a basis for predicting variation in growth phenology patterns and species-specific responses.

### Differences in the growth pattern of understory and canopy trees

The growing season of understory trees started later and ended sooner, with fewer growing hours during the growing season (in both absolute and relative number of growing hours; Figure 3) than canopy trees. Additionally, changes in growth dynamics of understory trees related to seasonal light availability were negligible (Figure 5 and Figure S8 available as Supplementary Data at *Tree Physiology* Online), whereas canopy trees exhibited gradual growth declines from the beginning to the end of the growing season. Data from Eustaška show that greater canopy openness increased the number of growing hours for understory trees (Figure S10 available as Supplementary Data at *Tree Physiology* Online), which is in agreement with studies pointing out that a dense canopy limits growth by reducing photosynthetic rates due to sub-saturating light conditions (Messier et al. 1998, Balandier et al. 2007, Liu et al. 2018, Strieder and Vospernik 2021). Despite intense solar irradiance limiting the growth of canopy trees at daily scales (Kašpar et al. 2024), numerous studies provide both direct (Wicklein et al. 2012, Xu et al. 2023) and indirect evidence (Splechtna et al. 2005, Šamonil et al. 2013, Kašpar et al. 2020, Facciano et al. 2023) linking available light to tree growth at annual scales. However, consistent with this, our results supported hypothesis (i) only partially: differences in growth onset between understory and canopy trees were not consistently significant across species and sites, and we did not observe a pronounced early-season growth peak. Instead, growth remained relatively constant throughout the growing season in understory trees. Thus, for the purpose of calibrating models that simulate forest carbon balance, it would therefore be valuable to investigate how variation in light availability shapes source–sink dynamics across gradients of tree size, canopy position and solar exposure.

Understory trees showed less variation in the proportion of growing hours across different day phases (Figure 4 and Figure S6 available as Supplementary Data at *Tree Physiology* Online). This suggests that conditions under the canopy mitigate the negative effect of high temperatures and solar radiation during the day, as previously reported by von Arx et al. (2013) and De Frenne et al. (2021). Although previous studies have reported higher probabilities of night-time growth (Zweifel et al. 2021, Salomón and Camarero 2025), our study supports this mainly for canopy trees, having higher growth probability during the night time, while understory trees showed more equal growth probability among individual growth phases (Figure 4). The more nuanced delimitation of night used in our study revealed a lower growth probability during sunset and a higher growth probability during sunrise compared with the full night phase (Figure 4 and Figure S6 available as Supplementary Data at *Tree Physiology* Online). Nevertheless, most of the growing hours (and growth) of canopy trees occurred during the day, reflecting the higher proportion of daylight hours over the growing season (42.7–58.9%; Figures 1D and 3C, D). The mitigating effect of the canopy resulted in understory trees realizing ~3.0–23.7% more growing hours and annual growth during the daytime than canopy trees (except for spruce in Eustaška), but overall, understory trees had fewer growing hours.

Our models show that climatic predictors explained less variability in the growth probability of understory trees (Figure 6). Still, VPD remained the strongest predictor of growth probability for both understory and canopy trees. While this has been already well documented for the latter (Cabon et al. 2020, Peters et al. 2021, 2023, Tumajer et al. 2022), our study confirms a weaker but still significant effect for understory trees. The mitigating effect of canopy also significantly reduced the effect of temperatures, as addressed by Rollinson et al. (2021), supporting hypothesis (ii) that the main canopy layer plays a critical role in buffering understory microclimate and reducing tree exposure to atmospheric stressors. This reduction is due to smaller differences in climatic metrics between growing and non-growing hours for understory trees (Figure S11 available as Supplementary Data at *Tree Physiology* Online). Understory trees tend to maximize CO_2_ exchange (Walters and Reich 2000), which makes them more susceptible to TWD under low light and leaving only small amounts of carbon (leftovers) available for growth (Palacio et al. 2014, Hartmann and Trumbore 2016, Gessler and Zweifel 2024). As a result, some climatically favorable periods remain unused for stem increment. Understory trees, in particular, experienced more TWD hours or hours with no change in stem dimensions. As trees grow larger, their increased stem mass and leaf area require higher maintenance costs (Olson et al. 2021). However, their canopy position and larger leaf areas ensure more consistent supplies of assimilates, leading to more balanced growth. Theoretically mitigated growth conditions for understory trees (von Arx et al. 2013, De Frenne et al. 2021) then result in their lower growth response to the meteorological conditions compared with canopy trees.

Although understory trees had fewer growing hours during the season, they exhibited a higher relative SDI than canopy trees at both the annual scale (Figure S5A available as Supplementary Data at *Tree Physiology* Online) and at the level of individual growing hours (Figure 4 and Figure S7 available as Supplementary Data at *Tree Physiology* Online). This can be attributed to their lower stem mass, as ontogenetic variation in biomass allocation and structural investment may result in higher relative growth in smaller individuals (similar effect applies TWD in the opposite way; Hérault et al. 2011). Nevertheless, in agreement with Etzold et al. (2022), the annual SDI of understory trees remained dependent on the number of growing hours in the growing season. The stochastic response of understory trees to climatic conditions led to a decoupling of annual growth increments between understory and canopy trees (Figure S12 available as Supplementary Data at *Tree Physiology* Online), supporting previous findings that understory trees exhibit lower climate–growth sensitivity than canopy trees (Facciano et al. 2023, Wang et al. 2023).

Our measurements revealed not only that understory trees experienced fewer growing hours but also that several individuals did not grow at all over the entire study period (Figure S2 available as Supplementary Data at *Tree Physiology* Online). The reduction of secondary processes (such as radial growth) helps shade-tolerant tree species survive prolonged sub-saturating light conditions (Williams et al. 1999). Beech can maintain primary (apical) growth when secondary growth ceases (Noyer et al. 2019), and spruce prioritizes primary growth when competing with dwarf mountain pine (*Pinus mugo* L.) for access to resources, particularly light (Šenfeldr et al. 2014, Kašpar et al. 2017). Secondary growth of understory trees is often minimal due to low light availability and competition, and may fall below the resolution of dendrometer measurements. Consistent with this limitation, we excluded data of 55 growing season of understory trees (1 in Boubín, 4 in Eustaška, 22 in Ranšpurk, 28 in Žofín) in which the pivotal dendrometers did not detect any measurable growth. It remains questionable how much understory trees, and certain species in particular, actually benefit from more convenient microclimatic conditions.

### Species-specific differences

We monitored tree species with contrasting shade tolerance: light-demanding maple and elm, intermediately shade-tolerant spruce, and shade-tolerant hornbeam and beech (Lichtenthaler et al. 2007, Petritan et al. 2007, Caudullo and De Rigo 2016, Sikkema et al. 2016, Zecchin et al. 2016).

Shade-tolerant tree species exhibited a lower proportion of growing hours during the understory stage, but this pattern reversed once they reached the canopy (Figure 3A). This can be illustrated by beech, which has low growth rates under the canopy and significantly increase growth rates after canopy opening compared with spruce or maple (Pavlin et al. 2024). Moreover, the high crown plasticity of beech compared with conifers enables more efficient canopy space filling, ensuring greater resource availability (Krůček et al. 2019). Additionally, the production of denser wood in beech and hornbeam (compared with spruce and maple or elm) requires greater carbon investments (Jandl et al. 2007, Hérault et al. 2011). These trait-based differences in growth result in a lower proportion of growing hours in beech, which also affects their absolute and relative growth increments (Table 1 and Figure 3). In canopy trees, contrasting patterns were observed, particularly in beech and spruce. Juvenile wood of spruce has a lower density than mature wood, while the opposite is true for beech (Gryc et al. 2008, 2011).

Differences in wood density may partially explain the lower predictive power of growth probability models for tree species with denser wood (e.g., hornbeam vs maple with elm and spruce vs beech, respectively). These findings support a broader hypothesis that functional traits, such as wood density and tree-crown plasticity, mediate species-specific growth responses to changing light conditions and tree social status in the forest. As a result, growth strategies are not fixed but shift throughout ontogeny, particularly during the transition from the understory to the canopy.

The strongest climate–growth relationship among all investigated species was identified for spruce, which also showed the least variability in growth probability between understory and canopy trees compared with other species. Spruce also showed the highest drought susceptibility (Kahmen et al. 2022), which is related to its shallow rooting depth, compared with beech (Schmid and Kazda 2002). This response may be linked to moisture limitations at the lower-elevated Žofín site, where drought frequently caused growth cessation in understory spruce (Figure 6 and Figure S13 available as Supplementary Data at *Tree Physiology* Online; Martín-Benito et al. 2008). At the higher-elevated Eustaška site (just below the treeline), our results identified heat limitation as a primary limiting factor (Figure 6; Treml et al. 2015). Differences in rooting depth between understory and canopy trees may represent an additional source of uncertainty in our results by potentially making understory trees more susceptible to drought conditions. However, in line with previous results (Carnwath et al. 2012, Martínez-Vilalta et al. 2012, Zang et al. 2012, Wang et al. 2023), our models indicated that drought has a stronger negative impact on canopy trees than on understory trees across all species.

### Methodological considerations

Monitoring tree growth across all forest vertical layers, from understory to canopy, is essential for quantifying ecosystem-level carbon dynamics. Although suppressed understory trees grow slowly, they contribute to carbon storage, turnover and competitive interactions that shape stand-level carbon allocation. Excluding lower layers can therefore bias carbon flux estimates and obscure mechanisms governing whole-ecosystem carbon balance. Progress will require integrating high-resolution growth measurements with 3D forest structure to develop digital twins that more realistically represent vertical carbon allocation. In the present study, we used a combination of band dendrometers, which measure tree circumference, and point (pivotal) dendrometers, which record stem diameter (DBH) at two fixed positions. Previous studies have shown that point dendrometers tend to capture higher-amplitude fluctuations, while long-term growth trends are generally comparable between the two instrument types (Corell et al. 2014, De Belder 2015). Ideally, studies investigating differences between trees of varying sizes should be based on the same measurement principle. However, due to limitations related to tree size and restrictions on the installation of monitoring equipment in protected areas, especially when invasive installation methods are required, using the same measurement principle for all trees is not always feasible. The setup used in this study is based on circumference measurements for canopy trees (recalculated to DBH) and on two-point measurements for understory trees (direct DBH measurement, more comparable with dendrochronological studies), which may represent a source of uncertainty, when some differences in the growth patterns of canopy and understory trees (e.g., higher autocorrelation in band dendrometers; Table S1 available as Supplementary Data at *Tree Physiology* Online, and a greater number of hours with no detectable stem change between consecutive measurements; Figure S4D available as Supplementary Data at *Tree Physiology* Online) may be related to differences in anatomical structure (cell size) and hormone concentrations arising from differences in pathway length (Sorce et al. 2013).

Despite substantial differences in temperature–precipitation regimes among study sites, and in growth patterns and climate sensitivity between canopy and understory trees, atmospheric conditions outweighed soil water potential as the dominant driver of growth (Tumajer et al. 2022).

## Conclusions

Our study showed that understory tree growth is not primarily limited by atmospheric conditions but by carbon availability, resulting in fewer but more intense growth periods. Despite buffered microclimatic conditions, understory trees did not fully utilize favorable periods for growth, suggesting that light limitation constrains carbon supply and weakens the coupling between growth and climatic drivers. Consequently, models relying primarily on atmospheric variables may overestimate the responsiveness of understory trees to climate variability. Future research should directly quantify light availability and carbon balance across canopy gradients and more explicitly incorporate vertical forest structure into models of tree growth.

## Supplementary Material

Supplementary_materials_tpag093

## Data Availability

The scripts used for data and figure preparation, as well as data analysis, are available on GitHub (https://github.com/spizik/DifferencesInGrowthOfUnderstoryAndCanopyTrees). The data used in this study are available at Zenodo (https://doi.org/10.5281/zenodo.18273434).
