# Supplementary material for: Growth patterns and climate sensitivity differ for understory and canopy trees
Source: Tree Physiol. 2026 Jul 9;46(8):tpag093. doi: 10.1093/treephys/tpag093 (PMC13477147; doi:10.1093/treephys/tpag093)
Supplement: Supplementary file 1 [file supplementary_materials_tpag093.docx]

**Supplementary Material**

to

**Growth patterns and climate sensitivity differ for understory and canopy trees.**

by

Jakub Kašpar^1^, Jan Krejza^2,3^, Jan Holík^1^, David Janík^1^, Růžena Janoutová^1,2^ and Kamil Král^1^

**Corresponding author:**

Jakub Kašpar

Department of Forest Ecology, Landscape research institute, Lidická 25/27, 602 00 Brno, Czechia

Jakub.kaspar@vukoz.cz

1 – Department of Forest Ecology, Landscape research institute, Lidická 25/27, 602 00 Brno, Czechia

2 – Global Change Research Institute of the Czech Academy of Sciences, Bělidla 986/4a, 603 00 Brno, Czechia

3 – Department of Forest Ecology, Faculty of Forestry and Wood Technology, Mendel University in Brno, Zemědělská 3, 613 00 Brno, Czechia

**Figure S1:** Differences in the timing of the growing season when thresholds of 2.5% or 5% of the annual increment at the beginning and end of the growing season are omitted. (A) Data of differences in start of the growing season (SOS); (B) data of differences in the end of the growing season (EOS).

**Figure S2:** Growing season lengths across individual years. (A) Ranšpurk; (B) Žofín; (C) Boubín; (D) Eustaška.

**Figure S3:** Differences in growth phenology between canopy and understory trees. Specifically, the Day of the Year (DOY) when the 10^th^, 25^th^, 50^th^, 75^th^ and 90^th^ percentiles of annual increment were reached. (A) maple in Rašpurk; (B) hornbeam in Ranšpurk; (C) elm in Ranšpurk; (D) spruce in Žofín; (E) beech in Žofín; (F) spruce in Boubín; (G) beech in Boubín; and (H) spruce in Eustaška. The boxes denote the 25^th^, 50^th^ and 75^th^ percentiles, while the whiskers illustrate 1.5 times the interquartile range. Statistically significant differences (p < 0.05) between the two groups are marked with an asterisk.

**Figure S4:** Proportions of individual growth phases within the growing season. (A) The proportion of all non-growing periods; (B) the proportion of Tree Water Deficit (TWD) periods; (C) the proportion of stem tissue refilling periods; (D) the proportion of periods with no change in stem diameter. The boxes represent the 25th, 50th, and 75th percentiles, while the whiskers illustrate 1.5 times the interquartile range. Statistically significant differences (p < 0.05) between the two groups are marked with an asterisk.

**Figure S5:** Stem increments and Tree Water Deficits (TWD) of understory and canopy trees during growing and TWD hours. (A) Growth rate; (B) TWD rate; (C) growth rate relative to DBH; (D) TWD relative to DBH. Statistically significant differences (p < 0.05) between the two groups are marked with an asterisk.

**Figure S6:** Differences between the realized and expected distributions of growing hours and realized growth across day phases. (A) Differences between the realized and expected distributions of growing hours; (B) Differences between the realized and expected realized growth.

**Figure S7** Differences in growth rates between understory and canopy trees, defined using the zero-growth approach. (A) maple in Rašpurk; (B) hornbeam in Ranšpurk; (C) elm in Ranšpurk; (D) spruce in Žofín; (E) beech in Žofín; (F) spruce in Boubín; (G) beech in Boubín; and (H) spruce in Eustaška. The boxes represent the 25th, 50th, and 75th percentiles, while the whiskers illustrate 1.5 times the interquartile range.

**Figure S8:** Growth rates related to realized annual Stem Diameter Increment (SDI; left panels – A-H) and to specific days of the growing season (right panels – I-P). (A) maple in Ranšpurk; (B) hornbeam in Ranšpurk; (C) elm in Ranšpurk; (D) spruce in Žofín; (E) beech in Žofín; (F) spruce in Boubín; (G) beech in Boubín and (H) spruce in Eustaška. Statistically significant differences in trends in the proportion of growing hours throughout the growing season of understory and canopy trees (p < 0.05) are marked with an asterisk.

**Figure S9:** Results of generalized mixed-effects models predicting the probability of tree growth and estimates of selected meteorological predictors. To simulate probability of growth of understory trees, air temperature and humidity measured under the canopy was used. (A) maple in Ranšpurk; (B) hornbeam in Ranšpurk; (C) elm in Ranšpurk; (D) spruce in Žofín; (E) beech in Žofín; (F) spruce in Boubín; (G) beech in Boubín; (H) spruce in Eustaška. In upper barplots, the variability explained by the random effect is represented by the empty portion of the bar. Statistically significant predictors are marked with asterisks.

**Figure S10:** Relationships between tree crown exposure and the number of growing periods. (A) Canopy openness of understory trees across individual sites; (B) the relationship between the proportion of growing hours and canopy openness in Eustaška.

**Figure S11:** Differences in climate metrics during growing and Tree Water Deficit (TWD) hours in canopy and understory trees. (A) Temperature measured at the meteorological station; (B) temperature measured under the canopy; (C) VPD calculated at the meteorological station; (D) VPD measured under the canopy; (E) global radiation measured at the meteorological station. The boxes represent the 25th, 50th, and 75th percentiles, while the whiskers illustrate 1.5 times the interquartile range. Panels (C) and (D) correspond only to the growing and TWD hours of understory trees.

**Figure S12:** Time series of annual increments in Diameter at Breast Height (DBH) in understory and canopy trees. (A) Maple in Rašpurk; (B) hornbeam in Ranšpurk; (C) elm in Ranšpurk; (D) spruce in Žofín; (E) beech in Žofín; (F) spruce in Boubín; (G) beech in Boubín; and (H) spruce in Eustaška. The boxes represent the 25th, 50th, and 75th percentiles, while the whiskers illustrate 1.5 times the interquartile range.

**Figure S13:** **Bootstrapped growth curves of understory and canopy trees, temperatures, and Soil Water Potential (SWP) over the study years**. (A) Maple in Rašpurk; (B) hornbeam in Ranšpurk; (C) elm in Ranšpurk; (D) spruce in Žofín; (E) beech in Žofín; (F) spruce in Boubín; (G) beech in Boubín; and (H) spruce in Eustaška. Confidence intervals represent the 95% probability range of the mean value.

**Table S1:** Coefficients of Generalized Linear Mixed-Effects Models (GLMMs) predicting the probability of tree growth based on climatic predictors and day phases. Tree groups are abbreviated in the table header**: (Can)** refers to canopy trees; **(Und A)** denotes understory trees with meteorological data from the open-area weather station; and **(Und B)** denotes understory trees with meteorological data measured under the canopy. **VPD** stands for vapor pressure deficit, and **SWP** for soil water potential. Species and site combinations are shown for each column b*lock (e.g.,* spruce *in* Žofín). Rows represent fixed effects included in the models.

| Fixed effect | Ranšpurk - maple | | | | Ranšpurk - hornbeam | | | | Ranšpurk - elm | | | | Žofín - Spruce | | | | Žofín - beech | | | Boubín - spruce | | | | Boubín - beech | | | | Eustaška - spruce | | | |
| --- | --- | --- | --- | --- | --- | --- | --- | --- | --- | --- | --- | --- | --- | --- | --- | --- | --- | --- | --- | --- | --- | --- | --- | --- | --- | --- | --- | --- | --- | --- | --- |
|  | Can | Und A | Und B | Can | | Und A | Und B | Can | | Und A | Und B | Can | | Und A | Und B | Can | | Und A | Und B | | Can | Und A | Und B | | Can | Und A | Und B | | Can | Und A | Und B |
| Intercept | **-3.83** | **-2.62** | **-2.58** | **-2.75** | | **-23.03** | **-21.42** | **-3.32** | | **-2.55** | **-2.6** | **-3.58** | | **-3.01** | **-2.92** | **-1.72** | | **-3.5** | **-3.47** | | **-1.74** | **-2.34** | **-2.42** | | **-1.26** | **-2.61** | **-2.52** | | **-3.62** | **-2.03** | **-2.18** |
| Temperature | **0.49** | **0.12** | **0.08** | 0.03 | | **-2.50** | -8.39 | **0.43** | | 0.11 | 0.05 | **-0.31** | | 0.12 | **0.26** | 0.02 | | -0.02 | -0.04 | | **-0.41** | **-0.07** | **0.1** | | **0.16** | 0.07 | 0.15 | | **0.70** | **0.55** | **-0.04** |
| VPD | **-4.01** | **-1.22** | **0.1** | **-2.62** | | **-4.47** | -13.76 | **-3.41** | | **-1.21** | **0.36** | **-4.55** | | **-1.29** | 0.03 | **-1.55** | | **-0.47** | **0.12** | | **-2.01** | **-0.43** | **0.09** | | **-1.62** | **-0.03** | **0.04** | | **-4.66** | **-1.67** | **0.07** |
| Solar radiation | **0.17** | **0.29** | **-0.22** | **0.46** | | -15.02 | -0.90 | **0.10** | | **0.61** | **-0.34** | **-0.19** | | **0.40** | -0.09 | **-0.79** | | **0.13** | **-0.34** | | **0.28** | 0.03 | **-0.27** | | **-0.64** | **0.12** | **0.09** | | **0.51** | **0.14** | **0.10** |
| Precipitation | **0.08** | **0.07** | **0.36** | **0.18** | | -17.05 | **13.49** | **0.05** | | **0.27** | 0.15 | **0.16** | | 0.03 | 0.13 | **0.51** | | **0.13** | **0.43** | | **0.05** | **0.03** | **0.41** | | **0.18** | **0.05** | **0.19** | | **0.04** | **0.07** | **0.40** |
| SWP | **-0.16** | **-0.24** | **0.68** | **-0.43** | | -0.45 | 0.26 | -0.05 | | **0.14** | 0.17 | **-2.36** | | -1.10 | -0.07 | **-0.25** | | **-0.29** | **0.23** | | **-0.48** | **-0.22** | -0.08 | | **-0.32** | **0.11** | **0** | | **0.49** | **0.41** | **0.09** |
| Day length | **1.03** | **0.40** | -0.02 | **1.55** | | **14.95** | 4.61 | **0.66** | | 0.04 | 0.00 | **0.76** | | **0.14** | 0.02 | **0.61** | | **0.45** | 0.09 | | **0.86** | **0.18** | 0.00 | | **0.81** | **0.17** | -0.05 | | **1.29** | **0.28** | **-0.22** |
| VPD : SWP | **0.18** | -0.05 | 0.05 | **-0.08** | | **1.04** | 0.07 | -0.01 | | **0.20** | -0.09 | **-4.17** | | -1.99 | **0.23** | **-0.25** | | **-0.51** | **0.61** | | **-0.67** | **0.08** | 0.09 | | **-0.55** | **0.01** | **0.05** | | **-0.16** | **0.2** | **0.35** |
| Day Length : SWP | **0.48** | **0.67** | **0.19** | **0.83** | | 0.35 | -0.04 | **0.41** | | 0.15 | -0.17 | **-0.06** | | **0.04** | **0.46** | 0.01 | | **0.13** | **0.83** | | 0.01 | **-0.15** | 0.11 | | -0.02 | **-0.02** | **-0.06** | | **0.03** | **0.03** | **0.57** |
| VPD : Day length | **0.94** | **0.14** | -0.04 | **0.64** | | **3.97** | **-0.08** | **0.55** | | **-0.48** | **-0.79** | **1.27** | | **-0.32** | **-0.74** | **0.33** | | **-0.31** | **0.24** | | **0.83** | -0.01 | 0.24 | | **0.42** | **0.06** | **0.01** | | **1.38** | **0.09** | 0.02 |
| Solar radiation : Day length | **-0.13** | **-0.13** | **-0.06** | **-0.08** | | 11.69 | **-3.17** | **-0.19** | | 0.04 | 0.04 | **0.08** | | 0.12 | 0.14 | **-0.06** | | 0.05 | 0.05 | | **-0.28** | 0.04 | 0.01 | | **-0.3** | -0.1 | 0.24 | | 0.01 | **-0.02** | **0.47** |
| Night | **-0.08** | 0.09 | **-0.8** | 0.05 | | 0.24 | -13.66 | **-0.11** | | -0.02 | **-0.39** | **-0.5** | | 0.21 | **-1.11** | **0.18** | | **0.6** | **-0.36** | | **0.24** | **0.34** | **-0.54** | | **1.06** | **0.14** | **-0.12** | | 0.01 | **0.29** | **-1.35** |
| Sunrise | -0.03 | **0.18** | -0.04 | 0.07 | | 0.17 | 0.47 | **-0.11** | | -0.08 | -0.13 | **0.07** | | **0.53** | -0.14 | **0.56** | | **0.83** | **-0.63** | | **0.84** | **0.31** | **0.18** | | **1.39** | **0.14** | **0.02** | | **0.25** | **0.44** | **0.19** |
| Sunset | 0.09 | 0.09 | **0.17** | **0.17** | | 0.22 | **12.97** | -0.02 | | -0.56 | **-0.51** | **-1.07** | | **-0.78** | **-0.22** | **-0.40** | | **0.21** | **-0.42** | | **-0.26** | **0.26** | -0.04 | | **0.57** | **0.1** | **0.02** | | **-0.37** | 0.04 | **0.26** |
|  |  |  |  |  | |  |  |  | |  |  |  | |  |  |  | |  |  | |  |  |  | |  |  |  | |  |  |  |
| Marginal R2 | 0.75 | 0.30 | 0.26 | 0.66 | | 0.16 | 0.16 | 0.71 | | 0.07 | 0.05 | 0.93 | | 0.30 | 0.23 | 0.58 | | 0.23 | 0.26 | | 0.63 | 0.10 | 0.13 | | 0.59 | 0.02 | 0.03 | | 0.84 | 0.34 | 0.29 |
| Conditional R2 | 0.78 | 0.37 | 0.34 | 0.68 | |  |  | 0.76 | |  |  | 0.94 | | 0.30 | 0.23 | 0.65 | | 0.26 | 0.29 | | 0.64 | 0.13 | 0.18 | | 0.64 | 0.03 | 0.06 | | 0.86 | 0.36 | 0.32 |
| Residual autocorrelation | 0.2 | 0.13 | 0.15 | 0.23 | | -0.03 | -0.03 | 0.23 | | 0.08 | 0.05 | 0.21 | | 0.01 | 0.03 | 0.2 | | 0.02 | 0.04 | | 0.38 | 0.14 | 0.13 | | 0.29 | 0.08 | 0.08 | | 0.31 | 0.2 | 0.17 |
